# Prescribing for People With a Personality Disorder

**DOI:** 10.1192/bjo.2024.596

**Published:** 2024-08-01

**Authors:** Arun Kuruppath, Anneke Muller, Alison Fergusson

**Affiliations:** Cumbria Northumberland Tyne and Wear NHS Foundation Trust, Newcastle, United Kingdom

## Abstract

**Aims:**

The primary aim was to identify areas where there may be a significant gap in following the NICE recommendations.

To compare how antipsychotic and benzodiazepine prescribing practice in Community mental health team, measures against the national prescribing practices as identified in the POMH-UK Quality Improvement Project (QIP) 12b.

**Methods:**

The medical secretaries were contacted and asked to provide a list of patients seen as outpatients between March–September 2021 who have a diagnosis of personality disorder.

As there were multiple psychiatrists working in a team the cases to include were taken evenly from each caseload.

**Results:**

The frequency of diagnosis of personality disorder was more likely in females (31/40). Most common personality disorder diagnosed was EUPD (88.5%) followed by mixed Personality disorder (11.5%).

Among sample of patients selected, around 75% were prescribed some psychotropic medication including 52.5% (21/40) who were prescribed an antipsychotic medication.

Around 47.6 % (10/21) of the antipsychotic prescriptions were a new recommendation. Out of all the antipsychotic medications prescribed, quetiapine was by far the most common antipsychotic prescribed followed by aripiprazole.

In 38% of cases where antipsychotics were prescribed specifically for the management of Personality Disorder a rationale was given. Predominantly they were prescribed to reduce mood instability and impulsivity, and to aid sleep. Furthermore, none of the rationales given was in line with NICE recommendation.

Only 3.8% (5/21) of those prescribed antipsychotics were given a written information about antipsychotic effectiveness in PD and a plan to reduce antipsychotic medication was documented in only 28.57% (6/21).

A comorbid diagnosis was present in 62.5% (25/40) of the patients and the most common one was complex PTSD. The frequency of antipsychotic prescription was higher in those with a comorbid diagnosis (57.1%) and 42.8% in those without a comorbid diagnosis. However, there were differences in comorbidities present for patients prescribed antipsychotics as compared with those not prescribed antipsychotics. Those on antipsychotics tended to have comorbid diagnoses on the psychosis, bipolar spectrum disorders and PTSD whereas those not on an antipsychotic tended to be on the depressive or anxiety spectrum.

The other psychotropic medications used were antidepressants and benzodiazepines.

**Conclusion:**

In general, the frequency of prescribing antipsychotic medication to patients with personality disorder in the community mental health teams across Cumbria (52.5%) appears to be lower than the national average (57%). However, the prescriptions did not meet the requirements set out by the NICE guidelines. A significant gap between the recommendations and practice was identified.

In 38% of cases, in which antipsychotics had been prescribed specifically for personality disorder there was a rationale given. Even when a rationale was given it was to treat intrinsic features of Personality disorder which is contrary to what NICE recommends. Only 3.8% of prescriptions were supported with written information on the efficacy of antipsychotics in personality disorder.

